# Treatment outcomes of 3D-printed custom and conventional mini-implant assisted rapid palatal expanders (MARPE)

**DOI:** 10.1186/s40510-025-00576-0

**Published:** 2025-08-25

**Authors:** Yash Sharma, Heeyeon Suh, Jonas Bianchi, Audrey Yoon, Heesoo Oh

**Affiliations:** 1 Arthur A. Dugoni School of Dentistry, Department of Orthodontics, University of the Pacific, San Francisco, USA; 2 Stanford University, Stanford, USA

**Keywords:** Maxillary expansion, Mini-implant assisted rapid palatal expanders, 3D-printed custom

## Abstract

**Background:**

Maxillary expansion has been a treatment of choice for correcting transverse skeletal discrepancies, especially in growing patients. For older patients, Mini-implant Assisted Rapid Palatal Expansion (MARPE) offers a promising treatment option. This study evaluates the treatment outcomes of Custom 3D-printed MARPE compared to Conventional MARPE (MSE-II).

**Methods:**

This retrospective study analyzed CBCT images from 42 patients aged 16 to 35 years, comparing measurements before (T1) and after (T2) expansion. The conventional (*n* = 21) and custom (*n* = 21) MARPE groups were matched with age and sex. Skeletal and dental changes were evaluated measuring twelve distances and four angles using Dolphin Imaging Software (Chatsworth, Calif). The measurements included frontozygomatic and maxillary widths, nasal cavity width, and dentoalveolar inclination. The effectiveness of each appliance was evaluated based on magnitude of expansion and successful correction of transverse discrepancy.

**Results:**

The custom MARPE group demonstrated comparable or greater increase in width across various anatomical landmarks to the conventional group. Logistic regression suggested a trend toward higher odds of successful transverse discrepancy correction with custom MARPE.

**Conclusions:**

Custom 3D-printed MARPE appliances may offer advantages in achieving skeletal expansion in older patients. Individualized appliance design and strategic mini-implant placement could contribute to effective treatment. However, further research is needed to evaluate long-term outcomes, cost-effectiveness, and potential complications to better guide appliance selection for each patient.

## Background

Maxillomandibular transverse discrepancies are prevalent, occurring in 8–23% of individuals, with a prevalence of approximately 10% in adults [1]. Maxillary expansion is a common orthodontic treatment that aims to resolve maxillary transverse deficiency (MTD). The amount of skeletal expansion achieved is related to the level of intermaxillary suture interdigitation. During the adolescent period, the mid-palatal suture becomes more tortuous with increasing interdigitation [2]. In adulthood, around the third decade of life, the suture is eventually obliterated and fused by calcified tissue [3]. Maxillary expansion is most effective between ages 13–15 and may remain effective up to 18 in females and 21 in males, though with more dentoalveolar than skeletal effects and less predictable outcomes in older patients [4, 5]. The implementation of mini-implants in expander appliances has made it possible to increase the chance of splitting the mid-palatal suture after interdigitation with fewer adverse dental side effects [6]. Recent studies have shown that mini-implant assisted rapid palatal expansion (MARPE) to be an alternative method in correcting MTD in late adolescence [6, 7]. 

**Fig. 1:**
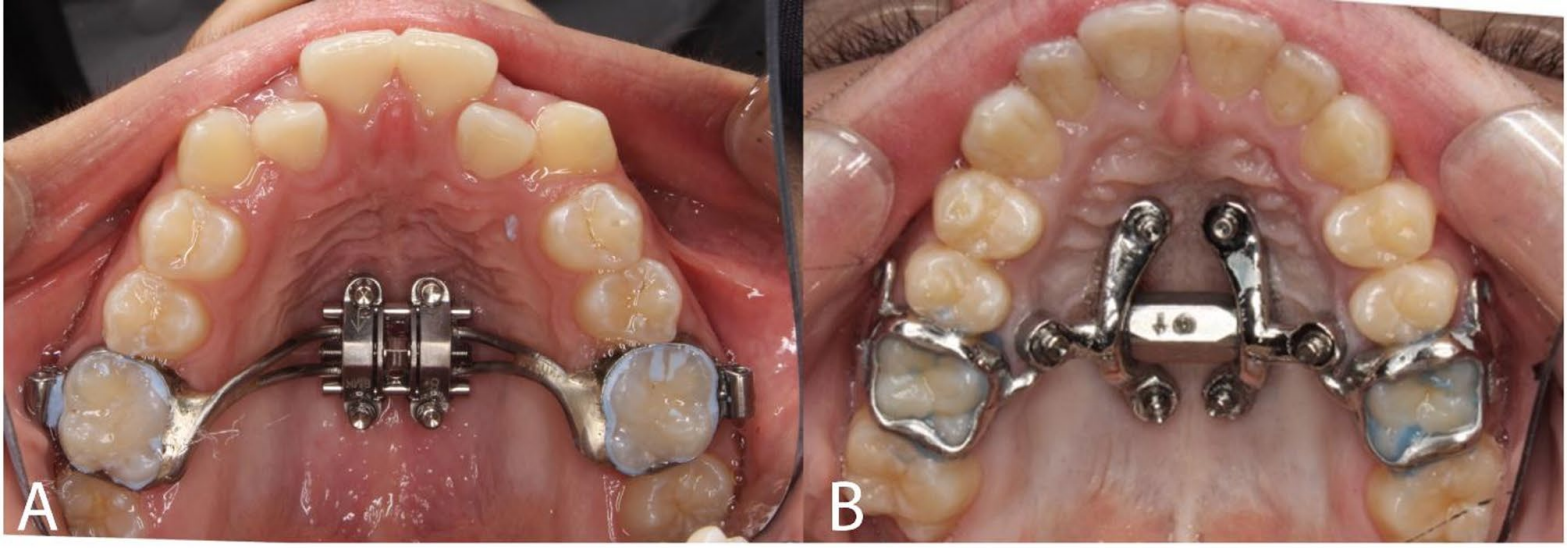
Two types of MARPE appliances use in the study. **A** Conventional MSE-II MARPE; **B** Custom 3D Metal Printed MARPE with TigerDental Powerscrew

In the past few years, there has been more of an emphasis on MARPE design and its effectiveness on maxillary expansion [8, 9]. Among the various designs, one that has been used widely is the MSE-II appliance (Maxillary Skeletal Expander Type II, Biomaterials Korea, Seoul, South Korea) (Fig. 1A). However, for patients aged 25 years and older, the success of the MSE II appliance diminishes significantly, and surgical assistance may be required to achieve the desired expansion [10]. The recent advancements in 3D metal printing technology and CBCT integration enabled precise virtual planning for mini-implant placements tailored to a patient’s palate, allowing for custom-fitted MARPE appliances that adapt to various palatal morphologies. However, research on this topic is still limited.

The purpose of this study is to evaluate the skeletal and dental changes after maxillary expansion using two different types of MARPE: the conventional MSE-II MARPE and the custom 3D metal printed MARPE. The null hypothesis is that there is no statistically significant difference in dental or skeletal changes, nor in the likelihood of achieving MTD correction, between the two types of MARPE used in patients with minimal growth potential.

## Methods

This study was approved by the institutional review board of University of the Pacific (#2020-74). The samples of this retrospective study were collected from three separate private practices (Bellflower, CA; Los Angeles, CA; Redwood City, CA) and one university clinic (University of the Pacific). A single clinician treated patients in the three private practices and oversaw the patients in the university clinic as a faculty member. The clinician used either type of MARPE for patients requiring at least 4 mm of skeletal expansion. A standardized protocol was implemented across all sites, including the acquisition of CBCT at pre-expansion (T1) and post-expansion (T2). The CBCT images were obtained using the i-CAT Dental Imaging System (Imaging Sciences International, Hatfield, PA) with a voxel size of 0.3 mm, 5 mA, 120 kV, and a field of view of at least 16 × 13 cm.

**Table 1:** Sample demographics

Category	Total number (Conventional: Custom)	%
*Clinic*		
A	15 (11:4)	36
B	15 (2:13)	36
C	10 (8:2)	23
D	2 (0:2)	5
*Sex*		
Male	32 (16:16)	76
Female	10 (5:5)	24
*Age (years)*		
16–20	12 (6:6)	29
21–25	10 (5:5)	24
26–31	14 (7:7)	33
> 31	6 (3:3)	14

**Fig. 2:**
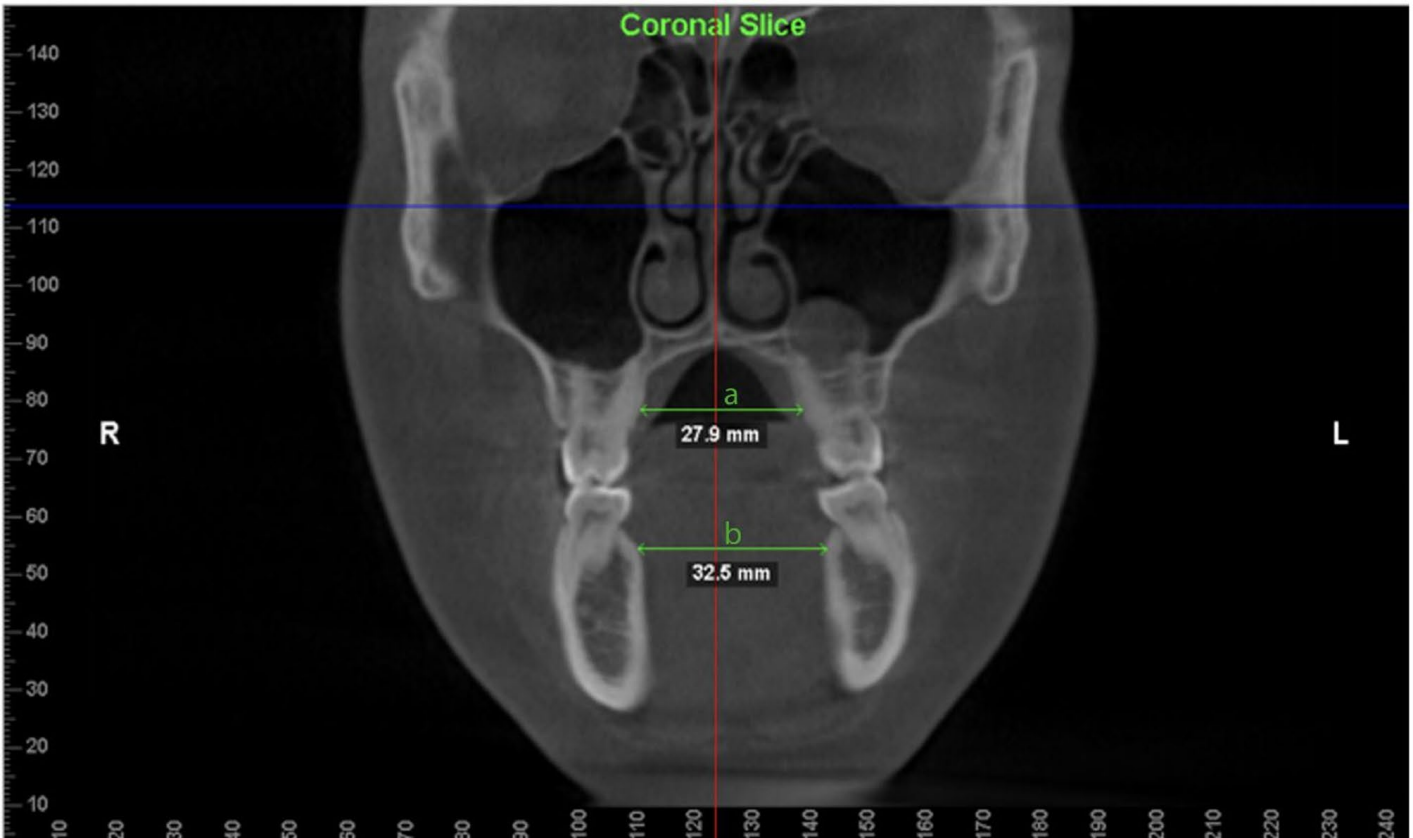
Maxillary and mandibular widths measured at the right first molar furcation level. **a** maxillary width (MxW, mm); **b** mandibular width (MnW, mm). Maxillomandibular skeletal transverse discrepancy (MxMnD, mm) can be calculated as (**a**–**b**)

The inclusion criteria were (1) patients who received MARPE due to maxillomandibular transverse discrepancy value less than 0 (Fig. 2), (2) CBCT records available of both pre- and post- expansion, (3) females > 16 years of age and males > 17 years of age. The exclusion criteria were (1) cleft lip and palate patients or other craniofacial deformities, (2) patients with diagnosed bone disorders, (3) No MARPE appliance in the oral cavity at T2 CBCT, (4) usage of other adjunctive orthopedic appliances (facemask, headgear, etc.), and (5) previous orthognathic surgery. The transverse discrepancy value was measured based on the method described by Miner et al., using the furcation level of the first molar root rather than the middle of the root for consistency in measurement [11]. 

The custom MARPE sample was compiled from consecutive patients with pre- and post- expansion records from April 2022 to July 2024. The conventional MARPE group was matched to the custom group based on age and sex. From a list of 103 consecutive conventional MARPE patients treated after 2017, patients were matched sequentially from the top of the list until the required number was reached. A total of 42 subjects (32 males and 10 females) were included in this study, with 21 patients in each group (Table 1).

**Fig. 3:**
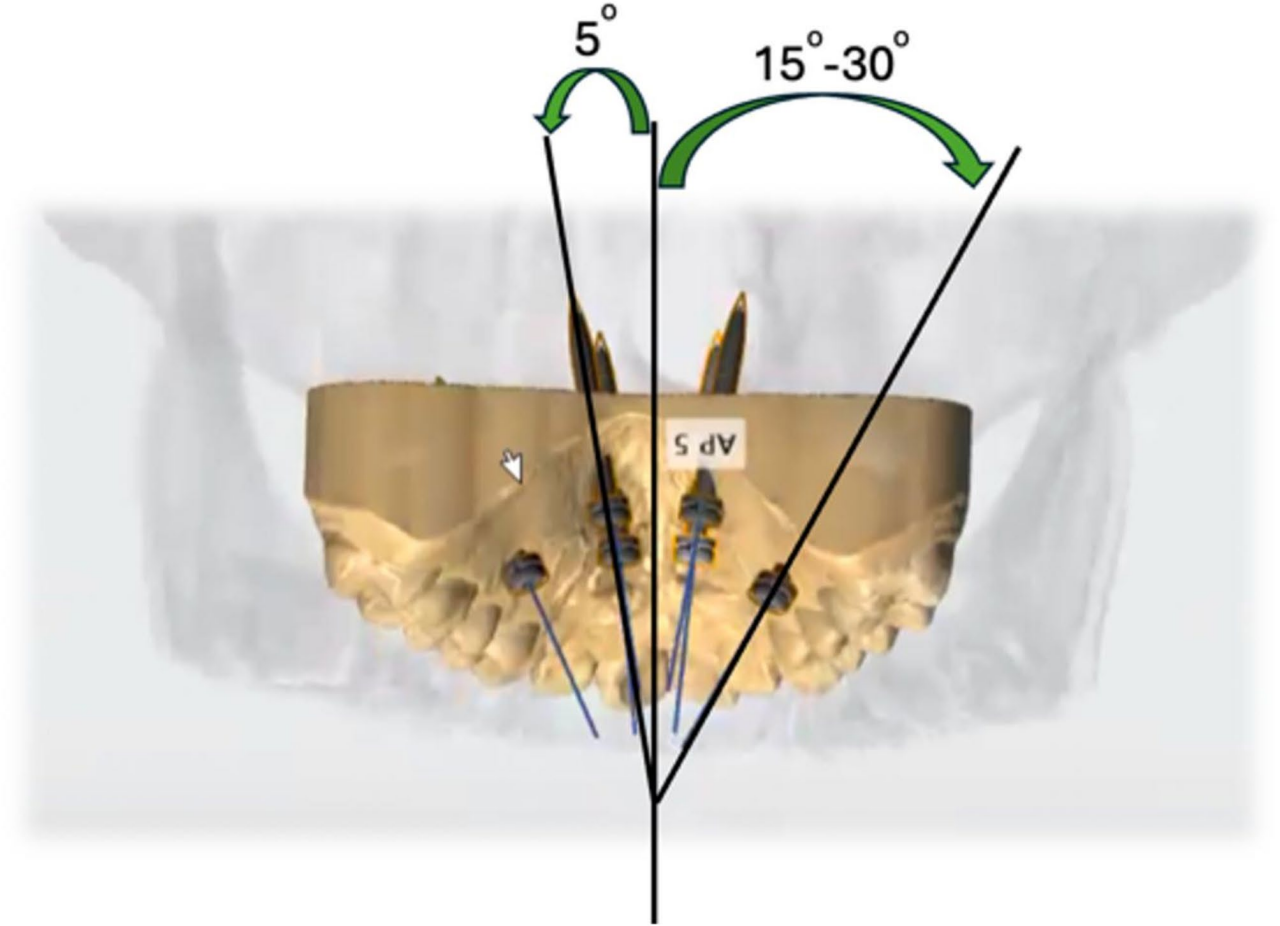
Mini-implant placement for custom 3D metal printed MARPE. 5° divergence lateral to the mid-palatal suture and 15–30° divergence between maxillary second premolar and first molar

MSE-II was used for the conventional MARPE and the custom 3D metal printed MARPE with Powerscrew (Tiger Dental, Bregenz, Austria) was used for the Custom MARPE (Fig. 1). The MSE-II design has a jackscrew centered to the palate with soldered arms connecting the main frame to the bands of the maxillary first molars. The prefabricated MSE-II base plate had 4 guides for placement of 4 mini-implants with the lengths of 9 mm, 11–13 mm and diameter of 1.8 mm. The custom MARPE was printed by Partners Dental Studio (Chanhassen, Minn). The custom MARPE had the Powerscrew jackscrew centered to the palate with arms connecting the main frame to the bands of maxillary first molars. The Custom 3D base plate had 6 or 8 guides for placement of 6 to 8 mini-implants with the length of 11 mm, 13 mm, 15 mm, or 17 mm and diameter of 2–2.5 mm. The base plate was customized to position mini-implants in areas of favorable bone density with proper divergence, and to ensure the jackscrew is well adapted to the palate. Four to six mini-implants were placed lateral to the mid-palatal suture at a 5-degree divergency, and 2 mini-implants were placed between maxillary second premolar and first molar at a 15–30 degree divergency (Fig. 3). For both the conventional and custom MARPE groups, the activation protocol implemented was two turns per day until successful sutural split and a slower expansion of $\:\le\:$ 1 turn per day until expansion is complete.

To evaluate skeletal and dental changes after expansion, distances and angles were measured in Dolphin Imaging Software (Dolphin Imaging & Management Solutions, Chatsworth, Calif). Initially, all images were orientated to three-dimensional planes: (1) Axial reference plane (Frankfort plane): defined by right and left orbitale, and right porion. (2) Mid-sagittal reference plane: a plane crossing Nasion and Basion perpendicular to the axial plane. 3) Coronal plane: a plane perpendicular to both the sagittal and axial planes. All records were anonymized prior to tracing and one evaluator (YS) responsible for CBCT measurements was blinded to the grouping. To assess intra-examiner reliability, 20 images were randomly selected. The evaluator repeated the measurements on these images after a 2–3 week interval from the initial assessment. The intraclass correlation coefficient (ICC) was then calculated.

**Fig. 4:**
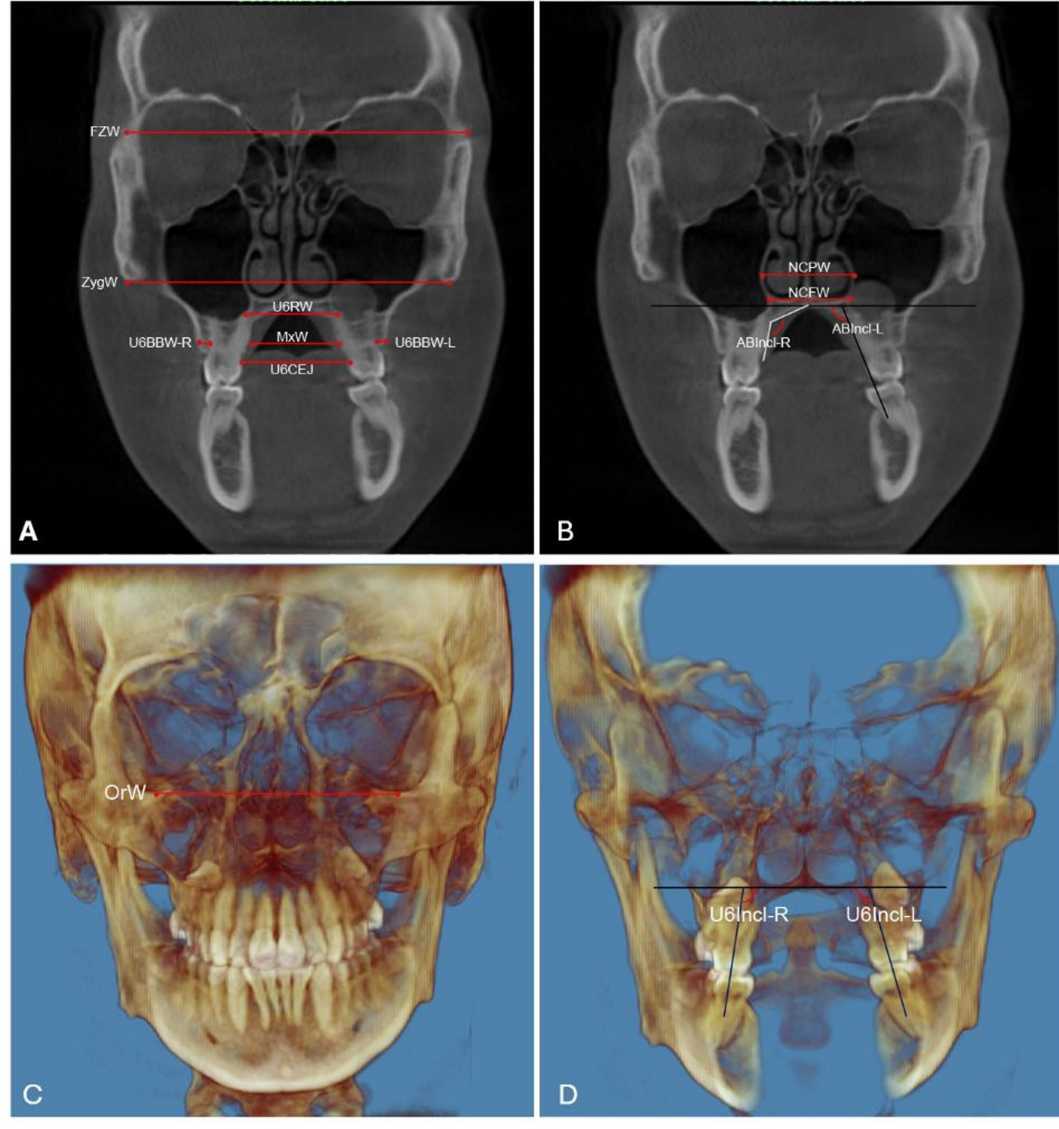
Skeletal and dental linear and angular measurements. **A** Frontozygomatic and maxillary linear measurements (mm); **B** Nasal cavity width (mm) and Dentoalveolar inclination (^o^); **C** Orbitale-Orbitale transverse width (mm); **D** U6 inclination (^o^); U6, maxillary first molar

**Fig. 5:**
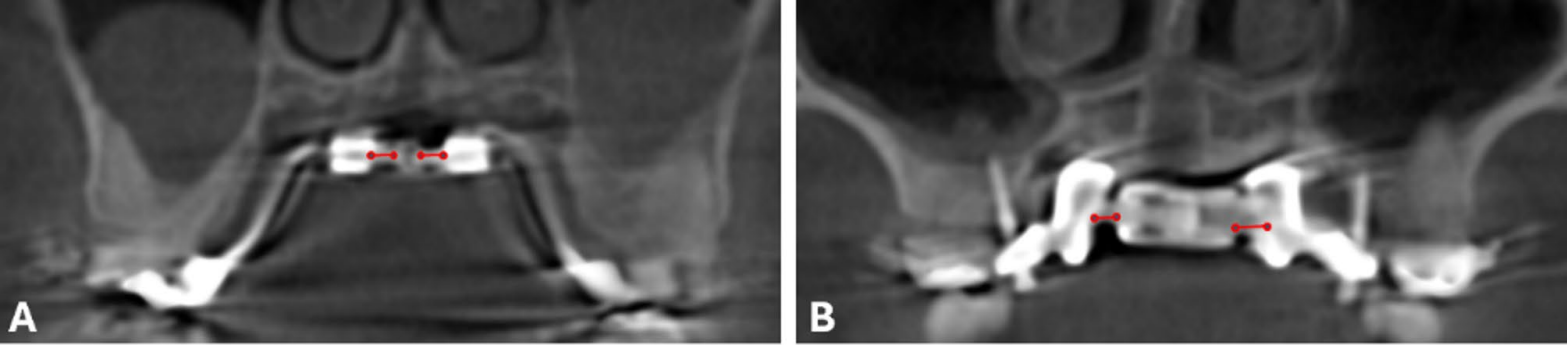
MARPE jackscrew expansion measurements on CBCT at T2. **A** conventional MARPE; **B** Custom MARPE

The outcome variables included in this study were 12 linear measurements and 4 angular measurements (Table 2; Fig. 4). At T2, the amount of jackscrew expansion was measured at the middle of the expansion screw by the sum of both sides of the expansion screw threads from the medial aspect of the lateral base plate to lateral aspect of jackscrew (Fig. 5). Maxillary skeletal expansion to screw opening ratio was evaluated by calculating the change in MxW, divided by the amount of screw opening (SCRW).

**Table 2:** Definition of the skeletal and dental measurements

Measurements		Definition
Expansion screw opening (SCRW, mm)		Sum of both sides of the expansion screw threads from the medial aspect of the lateral base plate to lateral aspect of hexagonal screw on the coronal section passing through center of expander palatal plate
Frontozygomatic Width (mm)	Fronto-Zygomatic Transverse Width (FZW)	Distance between most lateral portion of right and left fronto-zygomatic suture on the coronal section passing through U6 furcation bilaterally
	Orbitale-Orbitale Transverse Width (OrW)	Width from right to left orbitale on the coronal section passing through Orbitale bilaterally
	Base of Zygoma Transverse Width (ZygW)	Distance between the right and left most inferior point of zygomatic ridge on the coronal section passing through U6 furcation bilaterally
Nasal cavity Width (mm)	Nasal Cavity Posterior Width (NCPW)	The width of the nasal cavity measured from left pyriform aperture to right pyriform aperture
	Nasal Cavity Floor Width (NCFW)	Distance between the widest portion of the nasal cavity floor parallel to palatal plane
Maxilla (mm)	Inter-root apex Width (U6RW)	Distance between the right and left palatal root apices of U6s
	Maxillary Width (MxW)	Maxillary palatal bone width at the level of the furcation of the right first molar
	CEJ-CEJ Width (U6CEJ)	Width between cento-enamel junctions (CEJ) of U6s
Mandible (mm)	Mandibular Width (MnW)	Mandibular lingual bone width at the vertical level of the furcation of the right first mandibular molar on the coronal section passing through center of expander palatal plate
Buccal bone thickness (mm)	UR6 Buccal Bone Width (U6BBW-R/U6BBW-L)	Distance between right/left buccal cortical plate and right/left U6 buccal root at the vertical level of the right/left U6 furcation
Dentoalveolar inclination (^o^)	Alveolar Inclination (ABIncl-R/ABIncl-L)	Angle between the palatal alveolar bone and the palate at right/left U6
	U6 Inclination (U6Incl-R/U6Incl-L)	Angle between a reference line connecting right and left orbitale and a line drawn through the center of the crown and the furcation of right/left U6

*U6* maxillary first molar

The effectiveness of each MARPE appliance, as the primary outcome, was evaluated by (1) magnitude of maxillary expansion and (2) successful discrepancy correction. The magnitude of expansion was based on the amount of the MxW increase. Each individual case was evaluated and was allocated into three groups:

1. Minimal expansion: MxW increase < 2 mm.
2. Moderate expansion: MxW increase 2–4 mm.
3. Significant expansion: MxW increase > 4 mm.

Successful discrepancy correction was also used to evaluate the effectiveness of the treatment. If the maxillomandibular transverse discrepancy (MxMnD) is greater than 0 at T2, meaning the maxillary width is greater than the mandibular width, it is considered a successful correction of the discrepancy.

### Statistical analysis

A power analysis was conducted using G*Power (ver 3.1.9.7) with an alpha value of 0.05, a power of 0.80, and an effect size of 1, to detect 2 mm difference in maxillary expansion between two groups based on standard deviation value in the previous studies [12, 13]. The result indicated a sample size of 10 per group. The normality of the data was tested with the Shapiro-Wilk test. Treatment changes (T2-T1) between the custom and conventional MARPE groups were compared using independent t-tests. Logistic regression analysis was used to evaluate how the appliance type and initial MxMnD affect the success of MxMnD correction. P-values < 0.05 were considered statistically significant. All statistical analyses were performed using the R language (Vienna, Austria).

## Results

Intra-examiner reliability was excellent with ICC greater than 0.88 for all the measurements. All the patients had minimal to no growth potential with the mean age at T1 of 25.0 ± 6.0 years (Table 1). Approximately half of the patients were over 26 years old, and 76% of the sample were male. The mean time span between T1 and T2 CBCTs was 2.9 ± 2.2 months and 6.3 ± 5.1 months for conventional and custom MARPE groups, respectively.

At T1, initial MxMnD was comparable between two groups, − 3.7 ± 2.9 mm and − 3.2 ± 2.1 mm for conventional and custom groups, respectively (*P* = 0.58, Table 3). There were no significant differences between the groups in most measurements, except for two variables: OrW and ZygW. At T2, the MxMnD was − 1.1 ± 2.6 mm in the conventional MARPE group and 2.5 ± 2.4 mm in the custom MARPE group indicating a reduction in transverse discrepancy in both groups.

**Table 3:** Pre- and post-expansion measurements

	T1					T2				
	Conventional MARPE		Custom MARPE		*P*	Conventional MARPE		Custom MARPE		*P*
	Mean	Sd	Mean	Sd		Mean	Sd	Mean	Sd	
Expansion screw opening (mm)						5.63	1.32	6.40	1.35	0.072
Mx-Mn Discrepancy (mm)	−3.68	2.93	−3.23	2.06	0.575	−1.14	2.63	2.47	2.38	< 0.001
*Frontozygomatic Width (mm)*										
FZW	105.17	3.98	106.37	4.79	0.382	105.33	3.87	106.80	4.73	0.277
OrW	74.27	4.01	77.25	4.63	0.032	74.88	3.52	78.62	4.33	0.004
ZygW	89.88	3.92	94.04	6.40	0.015	91.19	4.04	97.29	5.73	< 0.001
*Nasal cavity Width (mm)*										
NCPW	30.80	2.98	30.57	3.04	0.803	32.29	3.15	34.26	3.52	0.063
NCFW	27.74	2.68	28.38	4.45	0.579	29.76	2.98	32.00	4.88	0.079
*Maxilla (mm)*										
U6RW	35.71	3.94	36.59	3.41	0.446	38.59	4.59	41.04	4.26	0.080
MxW	31.81	3.06	33.11	3.07	0.175	34.38	3.53	38.81	3.91	< 0.001
U6CEJ	34.18	3.75	35.82	3.44	0.146	38.72	4.43	41.80	3.75	0.019
*Mandible (mm)*										
MnW	35.55	3.12	36.35	3.10	0.409	-	-	-	-	-
*Buccal bone thickness (mm)*										
U6BBW-R	1.87	0.81	2.27	0.93	0.146	1.42	0.73	1.93	0.83	0.043
U6BBW-L	1.99	0.74	2.40	0.75	0.085	1.50	0.78	2.12	0.77	0.013
*Dentoalveolar inclination* (^o^)										
ABIncl-R	112.40	6.77	116.21	8.89	0.126	111.29	6.74	115.70	8.78	0.075
ABIncl-L	114.86	7.44	116.12	9.35	0.633	114.88	7.42	116.90	8.33	0.412
U6Incl-R	87.66	4.24	87.25	4.05	0.748	83.44	5.18	85.42	3.49	0.154
U6Incl-L	88.30	3.06	87.30	4.63	0.414	84.27	4.90	84.58	4.76	0.837

*P*, Independent t-test between Custom MARPE and Conventional MARPE groups; MnW was not measured at *T2*, as no treatment was provided on the mandibular arch between T1 and T2

The changes after expansion are presented in Table 4. The mean jackscrew separation (SCRW) was similar between the conventional and custom groups, measuring 5.6 ± 1.4 mm and 6.4 ± 1.4 mm, respectively. Both groups demonstrated significant maxillary expansion (MxW) following treatment (*P* < 0.001). The ratio of maxillary width increase to screw opening (MxW/SCRW) was 0.9 in the custom MARPE group and 0.4 in the conventional MARPE group (*P* = 0.0001).

**Table 4:** Pre- and post-expansion changes (T2-T1)

	Conventional MARPE			Custom MARPE			
	Mean	Sd	*P* ^a^	Mean	Sd	*P* ^a^	*P* ^b^
Expansion Screw opening (mm)	5.64	1.36		6.40	1.35		0.080
Mx-Mn Discrepancy (mm)	2.54	2.27	<0.001	5.70	1.95	0.000	<0.001
Mx expansion/screw	0.44	0.34		0.86	0.18		<0.001
*Frontozygomatic Width* (mm)							
FZW^c^	0.16	0.24	0.006	0.43	0.70	0.011	0.103
OrW^c^	0.61	0.76	0.001	1.38	2.52	0.021	0.189
ZygW	1.30	1.24	<0.001	3.25	2.16	<0.001	<0.001
*Nasal cavity width* (mm)							
NCPW	1.49	1.47	<0.001	3.70	1.49	<0.001	<0.001
NCFW	2.01	1.72	<0.001	3.63	2.09	<0.001	0.009
*Maxilla* (mm)							
U6RW	2.88	1.81	<0.001	4.45	2.71	<0.001	0.033
MxW	2.57	2.29	<0.001	5.70	1.95	<0.001	<0.001
U6CEJ	4.54	2.21	<0.001	5.98	2.18	<0.001	0.039
*Mandible* (mm)							
MnW	-	-	-	-	-	-	-
*Buccal bone thickness* (mm)							
U6BBW-R^c^	−0.44	0.41	<0.001	−0.34	0.27	<0.001	0.334
U6BBW-L^c^	−0.49	0.55	<0.001	−0.27	0.26	<0.001	0.104
*Dentoalveolar inclination* (^o^)							
ABIncl-R	−1.11	4.04	0.222	−0.50	6.22	0.714	0.710
ABIncl-L	0.02	4.67	0.985	0.78	7.10	0.620	0.683
U6Incl-R	−4.22	3.57	<0.001	−1.83	3.50	0.027	0.034
U6Incl-L	−4.02	4.43	<0.001	−2.71	4.09	0.006	0.326

^a^paired t-test between pre- and post-treatment measurements per group; ^b^ independent t-test comparing changes between Custom and Conventional MARPE groups; ^c^ Wilcoxon signed rank test

**Fig. 6:**
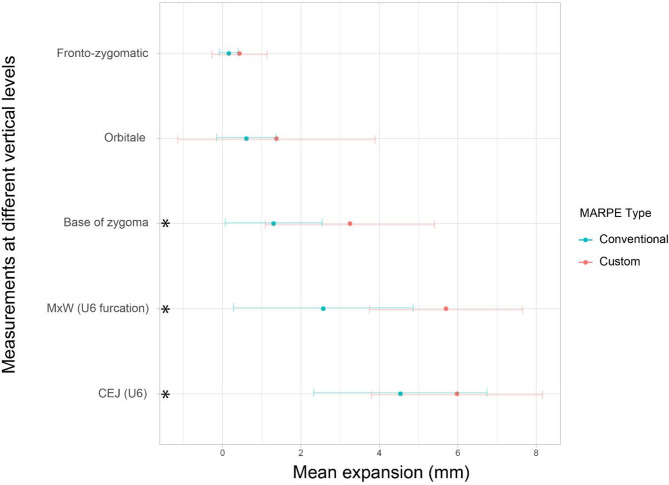
Mean expansion (mm) at different vertical levels. The error bars indicate the standard deviations. Blue, conventional MARPE group; red, custom MARPE group; MxW, Maxillary width; U6, maxillary first molar. *Indicates a statistically significant difference between conventional and custom MARPE groups

Both groups demonstrated statistically significant increase in transverse dimension at various anatomical landmarks. The custom MARPE group demonstrated a greater increase in transverse dimension at ZygW, U6RW, MxW, and U6CEJ compared to the conventional MARPE group, though considerable individual variability was observed in both groups (Fig. 6). The change in nasal cavity width, both NCPW and NCFW, was also statistically significant in both groups, with greater increase in the custom MARPE group.

**Fig. 7:**
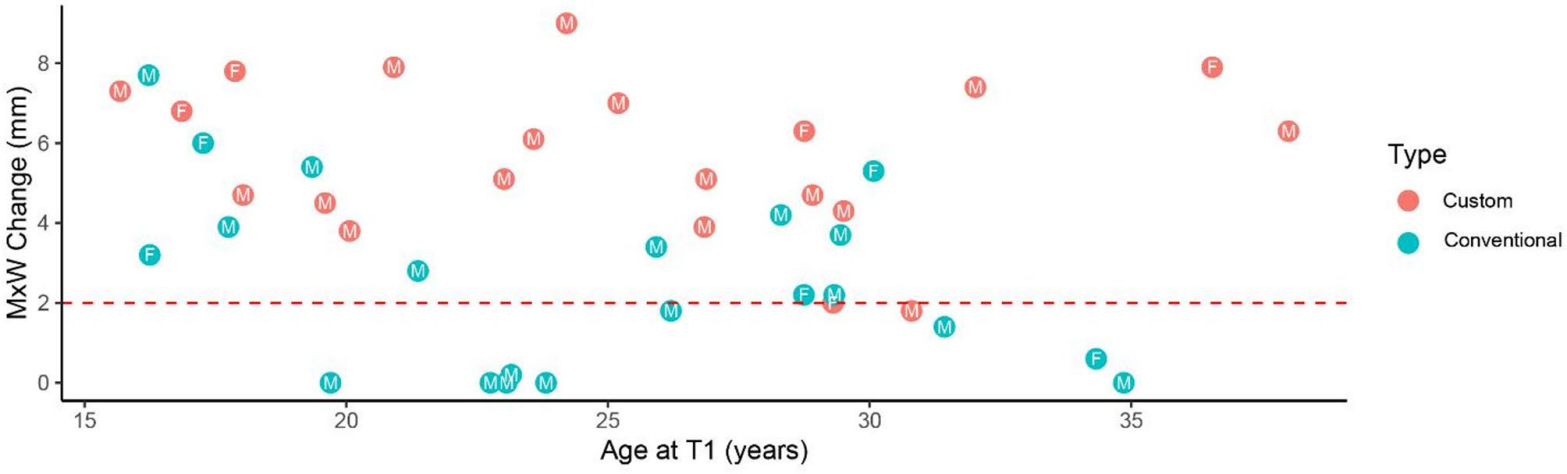
Scatter plot showing the relationship between age at T1 and maxillary width (MxW) change. Each point represents an individual patient, with the sex (M or F) labeled. Points are color-coded by MARPE type (Custom vs. Conventional). A red dashed horizontal line at 2 mm indicates the threshold for clinically meaningful expansion

Significant expansion (MxW increase > 4 mm) was achieved in 81% of patients in the custom MARPE group (Table 5). Among the nine conventional MARPE patients who did not achieve clinically relevant expansion, five were male patients over the age of 24, and one was a 35-year-old female patient. Six of these patients had an expansion of $\:\le\:$ 0.5 mm. Among the two custom MARPE patients who did not achieve clinically meaningful expansion, one was a 29-year-old female, and the other a 31-year-old male with a minimum expansion of 1.8 mm (Fig. 7).

**Table 5:** Magnitude of expansion

	*n* (%)	
	Conventional MARPE	Custom MARPE
Minimal expansion	9 (42.9)	2 (9.5)
Moderate expansion	7 (33.3)	2 (9.5)
Significant expansion	5 (23.8)	17 (81)

In correcting transverse discrepancy, 45% of patients in the conventional MARPE group and 80% in the custom MARPE group achieved successful transverse discrepancy correction. Logistic regression analysis (Table 6) suggested higher odds of successful correction with custom MARPE (OR = 8.7, *P* = 0.008). Logistic regression was adjusted for the amount of initial discrepancy.

**Table 6:** Odds ratio for successful maxillary transverse discrepancy correction depending on types of MARPE, adjusted by initial discrepancy amount

	OR	*P*-value	CI (95%)	
Custom MARPE vs. Conventional MARPE	8.71	0.008	1.74	43.61
|MxMnD|^a^ at T1 (mm)	0.66	0.022	0.46	0.94

*MxMnD*, Maxillomandibular transverse discrepancy

^a^ Absolute value

## Discussion

Several factors contribute to the success rate of MARPE, one key factor being the patient’s age [10]. The effectiveness of MARPE treatment in adolescents and young adults is well supported by numerous studies [12–16]. One study found a 0% failure rate of expansion in a sample population with an average age of 17.2 years (range: 14–26 years) [12]. Another study reported an 86.96% success rate of MARPE expansion in young adults with an average age of 20.9 ± 2.9 years [13]. Additionally, a negative correlation between age and mid-palatal suture opening with MARPE was observed in a group of young adults with an average age of 22.52 ± 5.11 years [16]. In this retrospective study, CBCT images of 42 patients with an age range between 16 and 35 years were used to analyze the anatomical changes with MARPE. The sample consisted predominantly of male patients (76%) and approximately half of the patients were over 26 years old, representing a group typically considered challenging to achieve significant skeletal expansion [15]. This study supports previous findings that MARPE can be effective for correcting MTD in late adolescence and young adulthood. Additionally, it shows promising outcomes with custom MARPE in an older population.

The design of the MARPE and placement of the mini-implants contribute to the success [8, 9, 17, 18]. Proper positioning of the mini-implants in areas with more favorable bone density and thickness ensures better anchorage and minimizes the risk of mini-implant loosening or failure [17, 18]. Another significant factor is the type of appliance used. Appliances with a more rigid jackscrew, as used in this study, which allow for more direct force transfer, have demonstrated higher success rates in achieving desirable skeletal changes [8, 9]. Custom MARPE may support treatment success in older population through individualized appliance design. The customized printing and jackscrew design allow for better adaptation to individual palatal morphology, positioning the force vector closer to the center of resistance of the dentomaxillary complex, which results in less rotation of the dentomaxillary complex in the frontal view [19]. Additionally, the custom design based on CBCT planning for mini-implant placement provide more anchorage points in areas with favorable bone density, potentially improving stability, especially in older patients. Also, a greater number of mini-implants in the custom MARPE group may allow for more force to be exerted for suture opening. However, the effects and potential complications of this increased force require further investigation.

A slower expansion protocol was implemented- two turns per day initially until successful suture split, followed by $\:\le\:$ 1 turn per day (1 turn: 0.17 mm for custom, 0.13 mm for conventional groups). Activation protocols for MARPE from previous studies have varied from once every other day [20] or once daily [15] (approximately 0.2 mm/day depending on the screw design) to four times daily [21] (1 mm/day), with the most common protocol being twice daily. A previous study suggested that a slower activation protocol might be necessary to dissipate stresses and prevent bone fractures around the mini-implants, which can cause loss of anchorage [22]. A previous randomized clinical trial in adolescents found that while both slow (0.1 mm/day) and rapid (0.5 mm/day) protocols were effective in correcting skeletal transverse maxillary deficiency, the rapid protocol resulted in more buccal tipping of maxillary molars, as well as mini-implant failures and bending [23]. Further study is needed to better understand how expansion protocols impact outcomes in older patients.

Our study did not find significant differences in dental changes between the custom and conventional MARPE groups. This outcome may be attributed to the inherent characteristics of MARPE, which distributes forces more evenly across the skeletal structures, reducing dental impacts.

This study has several limitations. First, restricting the sample to patients over the age of 20 and perfectly matching baseline characteristics was ideal but not feasible due to the retrospective study design. Second, the relatively short follow-up period may not adequately capture the long-term stability of the achieved expansion. Third, limitations in measurement include the inability to assess mid-palatal suture opening due to image resolution and remodeling that had already occurred. While MxMnD was measured at the first molar furcation to reduce the effect of dental tipping, alveolar bone remodeling cannot be completely ruled out. Therefore, success was conservatively defined as MxMnD ≥ 0 mm at T2 and clinically meaningful expansion as ≥ 2 mm, which may have underestimated success. Lastly, due to the retrospective nature of the study, data on quality of life, nasal airway function, and side effects such as pain or discomfort were not recorded. A prospective study would provide a more comprehensive understanding of these factors and the overall benefits of the treatment.

To the best of our knowledge, this is the first study to evaluate the outcomes of a 3D metal-printed custom MARPE compared to a conventional MARPE in patients with minimum growth potential. The results suggest that a personalized MARPE design, optimized for mini-implant placement, may enhance treatment outcomes. However, custom MARPE appliances tend to be bulkier with more mini-implants, which may affect speech, require a longer adjustment period, and lead to increased postoperative pain. They are also more costly than conventional options. Evaluation of potential complications, which may increase with age, along with treatment invasiveness and cost-effectiveness is needed to determine the most appropriate appliance for individual patients.

## Conclusions

In our study, custom 3D-printed MARPE appeared to be an effective alternative for patients with minimal growth potential, associated with greater maxillary skeletal expansion compared to conventional MARPE. Further research is needed to guide optimal appliance selection.

## Data Availability

The datasets used and/or analysed during the current study are available from the corresponding author on reasonable request.

## Abbreviations

MTD: Maxillary transverse deficiency
RPE: Rapid palatal expander
MARPE: Mini-implant assisted rapid palatal expansion
MxW: Maxillary width
MnW: Mandibular width
MxMnD: Maxillomandibular transverse discrepancy
ANS: Anterior nasal spine
PNS: Posterior nasal spine
